# Supplementary material for: A brain-wide map of neural activity during complex behaviour
Source: Nature. 2025 Sep 3;645(8079):177–91. doi: 10.1038/s41586-025-09235-0 (PMC12408349; doi:10.1038/s41586-025-09235-0)
Supplement: Supplementary file 1 — This file contains Supplementary Tables 1–4 and Supplementary Figs. 1–10. [file 41586_2025_9235_MOESM1_ESM.pdf]

nature portfolio

https://doi.org/10.1038/s41586-025-09235-0

Supplementary information

A brain-wide map of neural activity during complex behaviour

In the format provided by the authors and unedited

Supplementary information

filter	remaining			
	sessions	probes	neurons	regions
session and insertion QC	459	699	621,733	281
first wheel movement time & missing events	459	699	621,733	281
minimum 3 error trials	459	699	621,733	281
single unit QC	459	698	75,708	268
gray matter regions	459	698	65,336	266
minimum 5 units/region	455	691	63,357	245
minimum 2 sessions/region	454	690	62,990	210
minimum 20 units/region	454	689	62,857	201

Table 1. Session and probe filtering. The table indicates the progressive filtering of the sessions and probes based on the various inclusion criteria described in the text. The first row is based on the released dataset, while subsequent rows indicate data constraints associated with the various methods of analysis.

Decoded variable	Trial-relative time	Window start (ms)	Window end (ms)	Bin size (ms)	Regression type	Score	Null distribution
Stim side	Stim onset	0	100	100	Logistic	bal. acc.	pseudo-session
Wheel speed	first wheel movement time	-200	1000	20	Linear	R^2	imposter
Wheel velocity	first wheel movement time	-200	1000	20	Linear	R^2	imposter
Choice	first wheel movement time	-100	0	100	Logistic	bal. acc.	synthetic
Feedback	Feedback time	0	200	200	Logistic	bal. acc.	synthetic

Table 2. Details of decoding analysis. Decoded variables are the targets for regression. Spike sorted activity is summed within trial-relative bins and used as the regressors (Fig. S2b). Regression is performed with the specified regression type using L1 regularization and the cross-validation scheme described in the text. Performance is reported on held-out trials using the specified score, and this score is compared to a null distribution of the form listed to evaluate statistical significance.

Variable	Region	Analysis	Session eid	Neuron ID	N Neurons	BA/ΔR^2
Stimulus	VISp	Encoding	e0928e11-2b86-4387-a203-80c77fab5d52	598	-	
Stimulus	VISp	Decoding	e0928e11-2b86-4387-a203-80c77fab5d52	-	40	0.80
Choice	GRN	Encoding	a7763417-e0d6-4f2a-aa55-e382fd9b5fb8	80	-	
Choice	GRN	Decoding	671c7ea7-6726-4fbe-adeb-f89c2c8e489b	-	68	0.86
Feedback	IRN	Encoding	a7763417-e0d6-4f2a-aa55-e382fd9b5fb8	359	-	
Feedback	IRN	Decoding	e012d3e3-fdbc-4661-9ffa-5fa284e4e706	-	39	1.00
Wheel speed/velocity	GRN	Decoding	671c7ea7-6726-4fbe-adeb-f89c2c8e489b	-	68	$0.73/0.72$
Wheel speed/velocity	All	Encoding	-	-	64299	

Table 3. Session and neurons used for example encoding and decoding analysis. For the stimulus (Fig. 4), choice (Fig. 5), feedback (Fig. 6), and wheel speed/velocity (Fig. 7) we included figure panels showing example encoding and decoding analyses. Here, we list the variable name, region, and analysis type along with the session eid, encoding neuron ID number, decoding number of neurons and balanced accuracy (BA) or ΔR^2 improvement (for speed/velocity).

First name	Middle	Surname	Conceptualization	Data Curation	Formal Analysis	Funding Acquisition	Experimental investigation	Methodology	Project Administration	Resources	Software (pipeline development)	Supervision	Validation	Visualization	Writing - Original Draft Preparation	Writing - Review & Editing
Dora		Angelaki	++			++			+		+					
Brandon		Benson			+++			+	+		++		++	+	+	+
Julius		Benson		+			+++	+								
Daniel		Birman									+			++		++
Niccolò		Bonacchi	+	+++	+	+	+	+++	+++	+++	+++	++	+++	+	+	+
Kcenia		Bougrova		+			+									
Matteo		Carandini	+++			+++		++	++	+++		++		+++		+++
Joana	A	Catarino		++			+++									
Gaelle	A	Chapuis		+++		+		++	+++	+++	+	+++	++		+	+
Anne	K	Churchland	+++			+++		+		++		++				+
Yang		Dan							+	+		+				
Felicia		Davatolhagh		+++			+++	+								
Peter		Dayan	+++		+++	++	+	++	+++			+++			++	+++
Eric	EJ	DeWitt	++		+	+	++	++	++			+	+			+
Tatiana	A	Engel	++			++		++	++	++		+++			+++	+++
Michele		Fabbri		++							+++					
Mayo		Faulkner		+++							+++					
Ila	Rani	Fiete	+++		+++	+++		+++	+++	+++		+++	++	+++	+++	+++
Charles		Findling	++		+++			+++			++		+		+	
Laura		Freitas-Silva		+												
Berk		Gerçek	++		+++			+++			++		+++	+++	++	++
Kenneth	D	Harris	+++	+	+++	+++		+++	+++	+++	++	+++	+	+	+++	+++
Michael		Hausser	+++			+++		+	++	+++		++				+
Sonja	B	Hofer	+			+	+		+	+		+				
Fei		Hu		++			+++	+								
Félix		Hubert			+						+		+			
Julia	M	Huntenburg		+++							+++					
Christopher		Krasniak		+++			+++									
Anup		Khanal		++			+++	+					++			
Christopher		Langdon			+++			+++			++		++		++	++
Christopher		Langfield		+++							+++		++			
Petrina	Y P	Lau		++			+++	+	+	+	+	+	+			
Zachary	F	Mainen	+++			+++		+	+	+		++				++
Guido	T	Meijer		+			+++				+					
Nathaniel	J	Miska	+	+			+++	+					+			
Thomas	D	Mrsic-Flogel	+			+	+	+	+	+		+				+
Jean-Paul		Noel		++			+++	+								
Kai		Nylund			+									+		
Liam		Paninski				+++						++				+
Alejandro		Pan-Vazquez		++			+++	+								
Alexandre		Pouget	+++		++	+++		+++	++			+++	+	+	+	++
Cyrille		Rossant									+			++		
Noam		Roth		+++	+		+++	+	+		++			+		
Rylan		Schaeffer			+											
Michael		Schartner	++	+	+++			++			++		++	+++	++	++
Yanliang		Shi		+	+++			++			++		++	++	++	+
Karolina	Z	Socha	+	++			+++	+++	+	+	++	+	++			
Nicholas	A	Steinmetz	++	++	+	+		++		+	++	++	++	+	++	++
Karel		Svoboda	++	++		++		++		+		++	+			
Anne	E	Urai		+			++	+								
Miles	J	Wells									+++					
Steven	J	West		+	+			+		+	+		+			
Matthew	R	Whiteway		++	+++			++			++				++	++
Olivier		Winter		++				+			+++		+			
Ilana	B	Witten	+			+			+	+						

Table 4. Low (+), medium (++) and high (+++) author contributions.

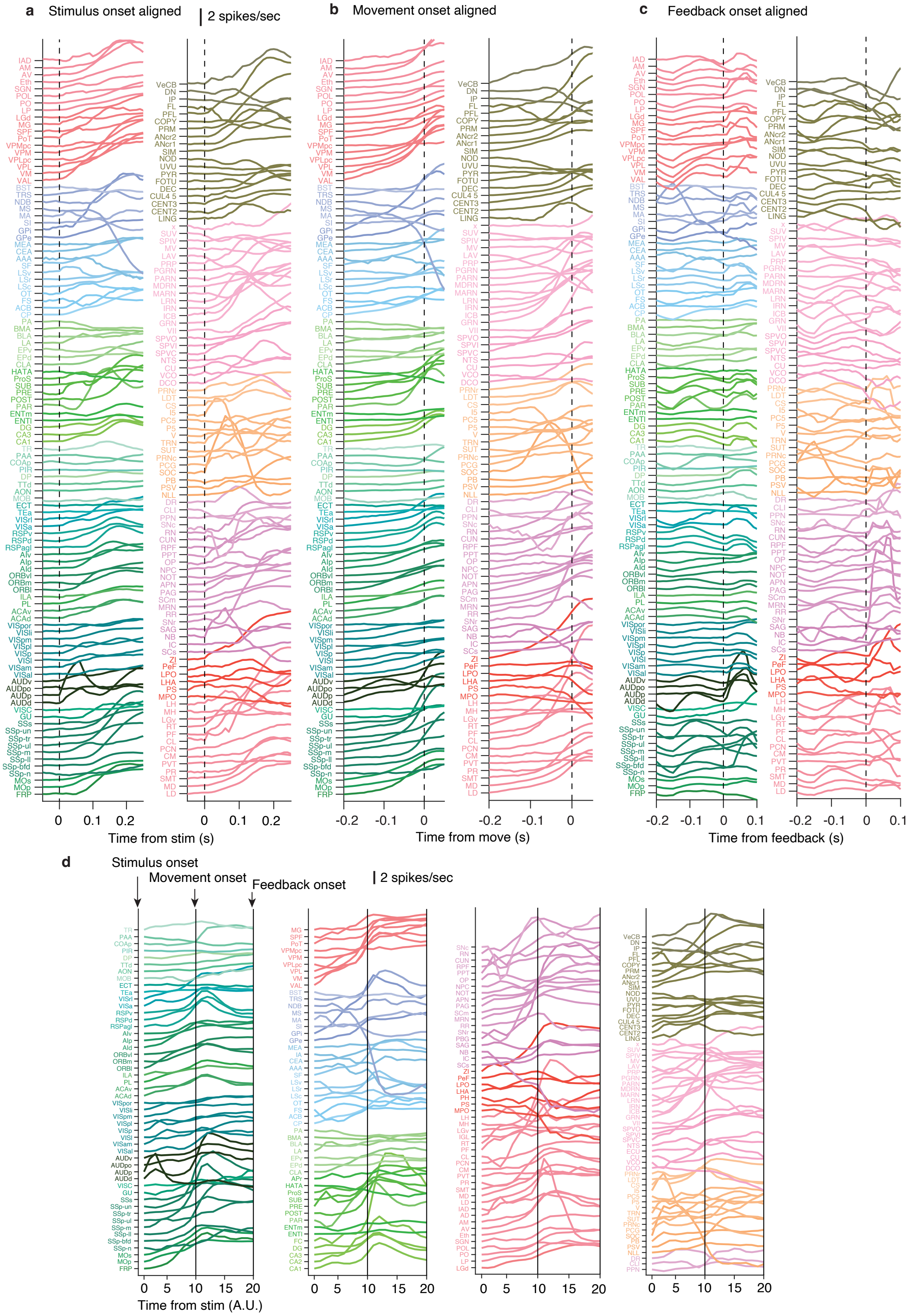

Figure S1. PSTH of average neural activity across the brain. **a)** PSTH of average neural activity aligned to stimulus onset, **b)** first wheel movement time, and **c)** Feedback onset. **d)** Time-warped PSTH of average neural activity across the brain. The duration between stimulus onset and first wheel movement time is divided into 10 equal-size time bins, as is the duration between onset to first wheel movement time and feedback onset for each trial (thus the length of time bin varies trial by trial). The spike rate of each time bin is computed and averaged across trials and sessions. This approach ensures stimulus, movement, and feedback onsets are perfectly aligned across trials.

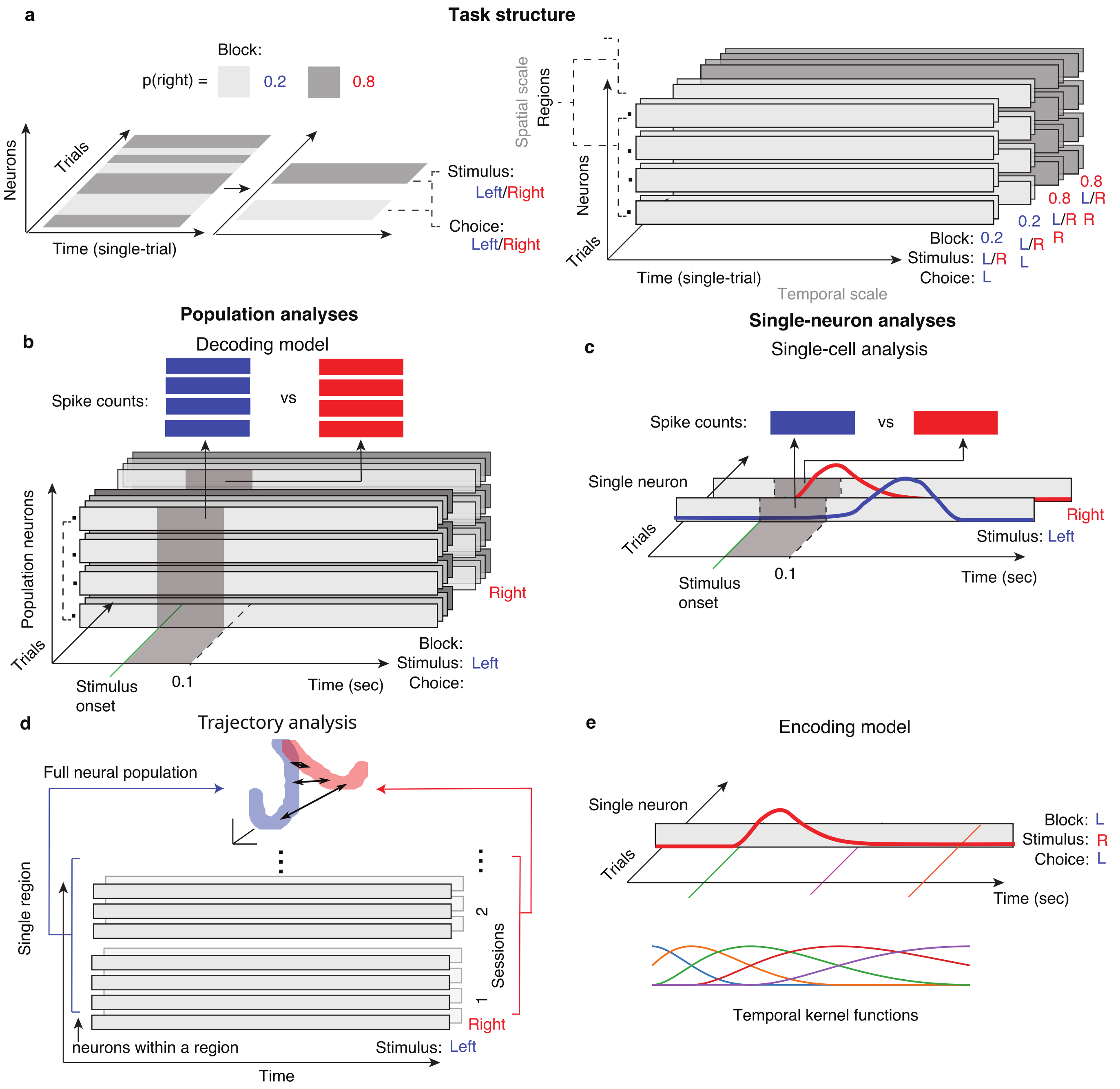

Figure S2. Detail of spatiotemporal structure of neural analyses. a) Task structures. In each session, consecutive trials form a block structure with the probability of a right-side stimulus being 0.2 and 0.8. In each block, there are trials with stimulus and choice side that are left or right. By regrouping trials, we can obtain 8 categories of trials with different combinations of stimulus side, block identify, and choice side. **b)** The decoding model studies the what can be extracted from the population neural activity about individual task variables within short time windows (without marginalizing other variables). **c)** Single-cell analysis studies the modulation of single-neuron activity by individual task variables within a short time window. **d)** Population trajectory analysis combines neural responses across multiple sessions (each neuron averaged across trials within a session) and analyses the trajectory of population neural activity. **e)** The encoding model uses temporal kernel functions to describe single-neuron activity during the entire trial at high temporal resolution.

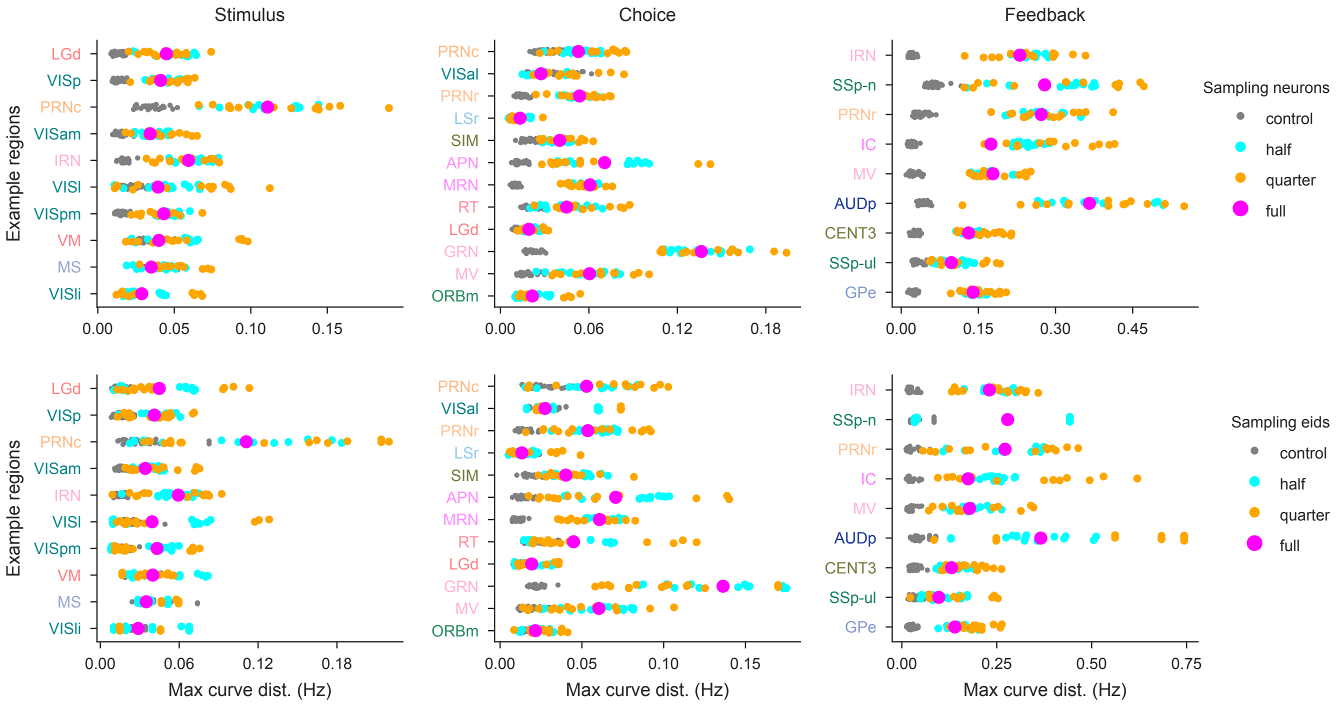

Figure S3. Maximal Euclidean distances for example regions with random data subsets. Each plot shows maximal population trajectory distances for the same highlighted regions as in the main figures (4l, 5l, 6l). The distances are computed after 15 subsamplings from half or a quarter of all neurons (top row of panels) or sessions (bottom row of panels). Grey dots indicate control scores for the sampled data, from trial randomization as in the main population trajectory analysis. For both, sampling neurons or sampling sessions, and most regions, the mean of the sampled scores visually matches the scores of the full dataset, showing that the strongest regional differences are also present for subsets of the data, however with more variance.

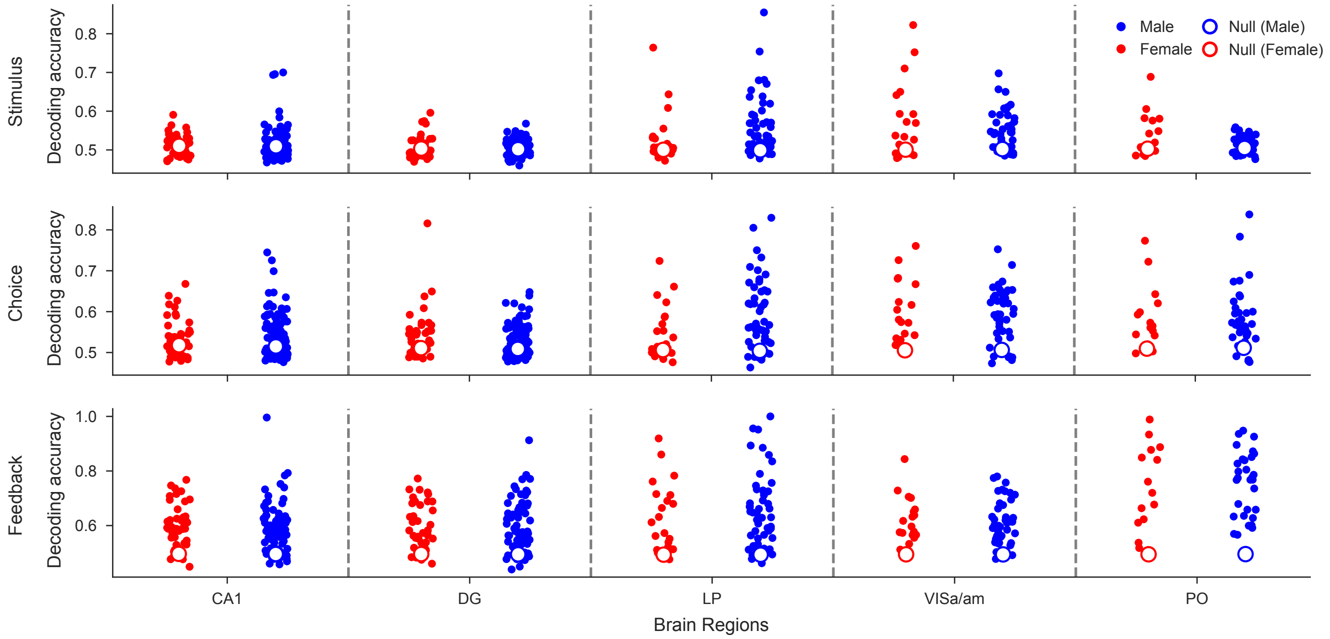

Figure S4. Decoding scores split by sex of mice For the 5 brain regions of the repeated site and our three main variables, decoding accuracies are shown, split by the sex of the mice. There is no obvious difference between the distributions. The total number of unique animals shown is 104, of which 69 are male.

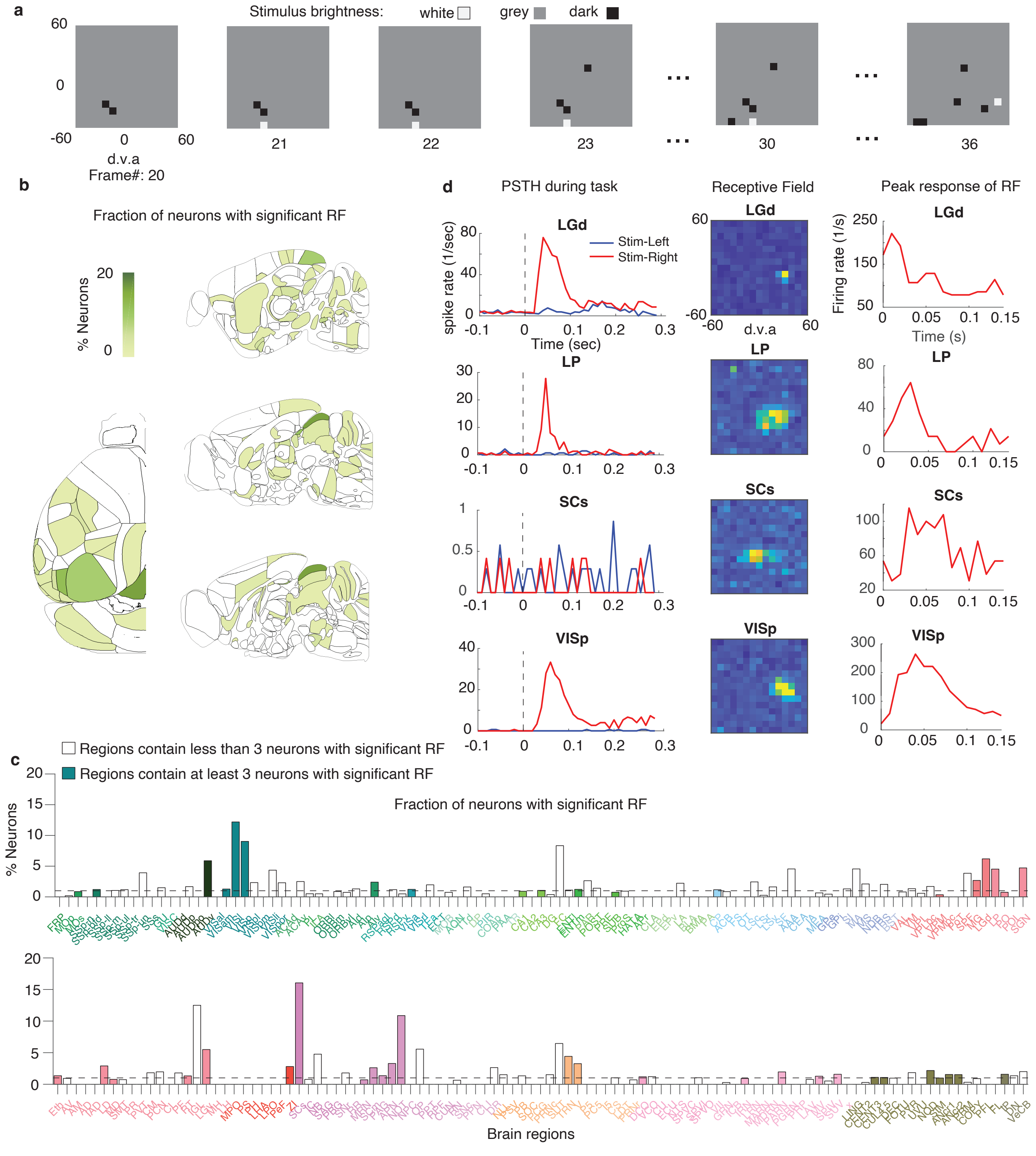

Figure S5. Receptive field mapping of single-cell across the brain a) Examples of the sequence of visual stimulus for receptive field mapping. Frame rate is 60 Hz. White/grey/dark pixels indicate white/grey/dark stimulus, respectively. (d.v.a. stands for degrees of visual angle) **b,c)** Fraction of neurons with significant receptive field in each brain region. In panel **c)**, the hollow bars indicate regions containing fewer than 3 neurons with significant RFs, while the filled bars indicate regions containing at least 3 neurons with significant RFs. **d)** PSTH during the task, the shape of the receptive fields, and the peak response of the receptive fields aligned to stimulus onset for example single cells with a significant receptive field. The peak response of the receptive fields is defined as the PSTH of the pixel in the receptive field with a maximal average spike rate.

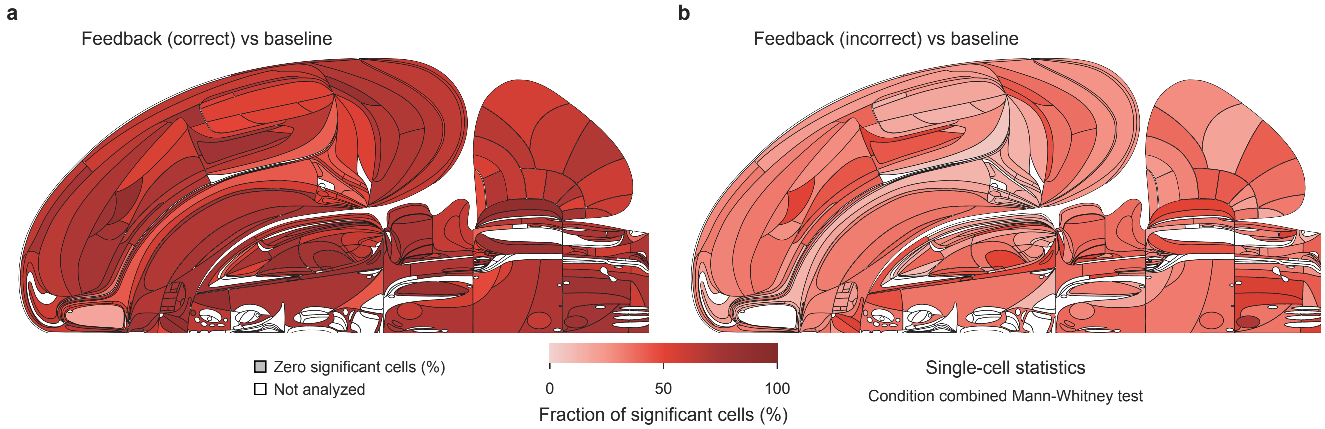

Figure S6. The modulation of neural activity by the feedback signal across the brain relative to baseline. a) Fraction of significant neurons per region identified by the condition combined Mann-Whitney test. We compared neural activity after correct feedback ([0, 200] ms) with baseline inter-trial neural activity ([-200, 0] ms aligned to stimulus onset). We deemed a region significant if the number of significant neurons there exceeded the $(1 - \alpha)$th percentile of a binomial (N, α) distribution (α=0.001), using $FDR_{0.01}$ to correct for multiple comparisons. (Methods). **b)** Comparison between neural activity after incorrect feedback ([0, 200] ms) with baseline inter-trial neural activity ([-200, 0] ms aligned to stimulus onset).

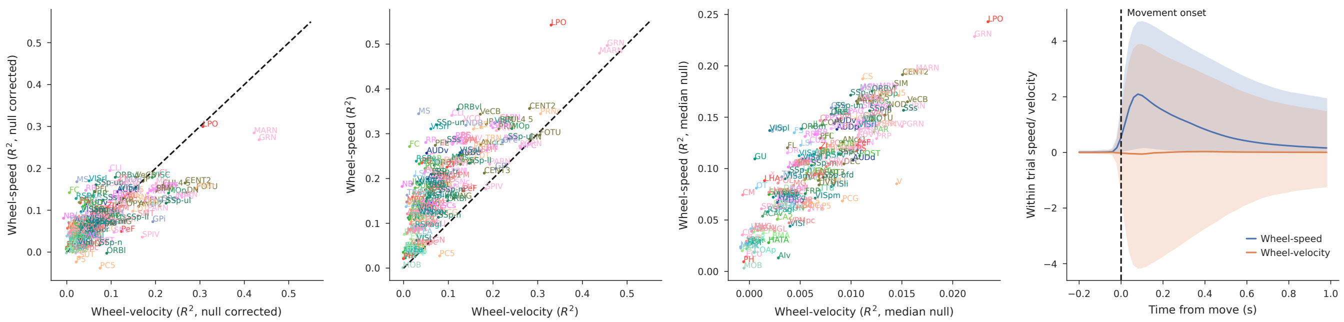

Figure S7. Decoding wheel-velocity versus wheel-speed. Scatter plots comparing per-region wheel-velocity decoding results against wheel-speed for all canonical regions. Decoding is performed for all session-region pairs in the canonical set and the median metric of all such pairs in a given region is plotted. Three such metrics are shown: **(a)** R^2 scores corrected by the median of the null distribution, **(b)** R^2 scores, and **(c)** median of the null distribution. Note the difference in scales for the axes in c. **(d)** The median wheel-speed and wheel-velocity trajectories across all trials are shown and the 5th to 95th percentiles are lightly shaded. The stereotyped shape of wheel-speed produces higher R^2 scores for sessions and null sessions. For example, computing R^2 between 400 randomly chosen trials and 400 repetitions of the median wheel-speed trajectory gives an R^2 of 0.244 (averaged across 1000 repeats). The same computation for wheel-velocity yields $R^2 = 0.000$.

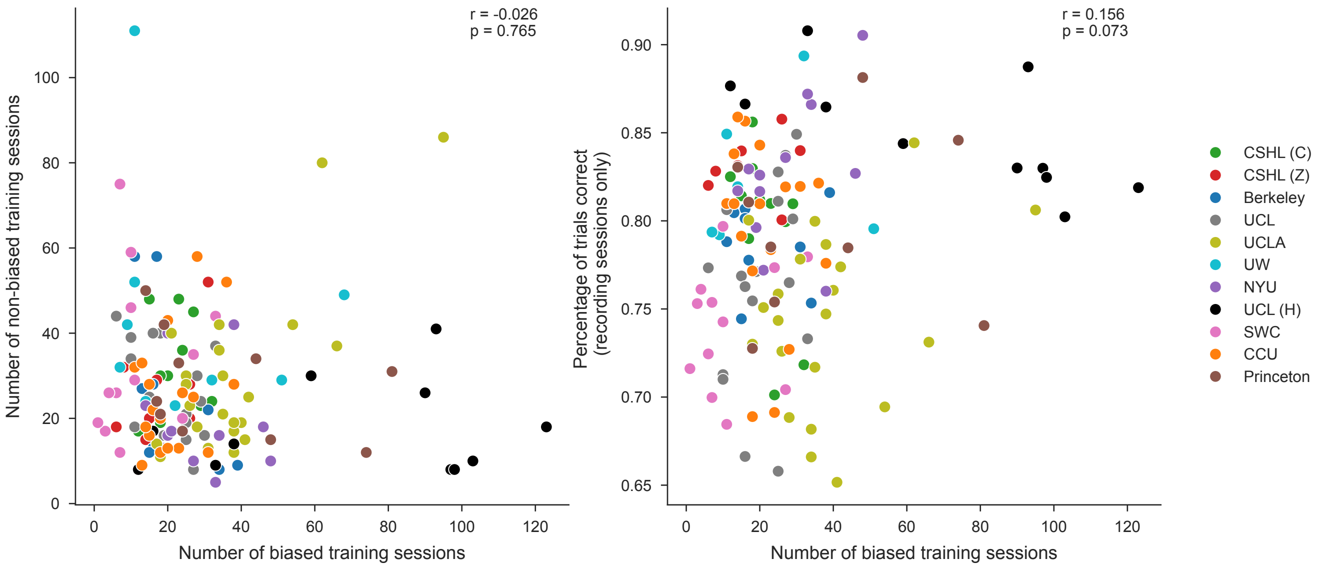

Figure S8. Training and performance statistics for each mouse, colored by lab, and their correlations across animals. Left: Scatter-plot of the number of biased versus unbiased training sessions of each animal. There is no significant correlation between these stages of training. **Right:** Scatter-plot of the number of biased training sessions of each mouse, against the overall percentage of correct trials across all recording sessions of that animal. There is again no significant correlation. The correlation between the number of pre-bias sessions and performance during recording is $r = -0.142, p = 0.094$ (not shown).

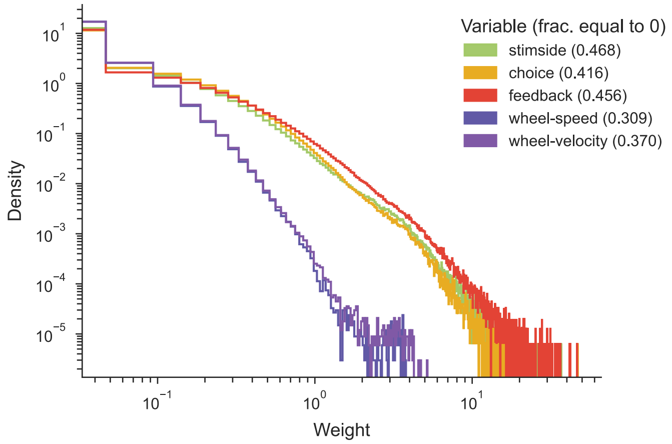

Figure S9. Histogram of regressor weights in decoding analysis. The distributions include decoding weights across all region-session pairs in the canonical set. The distribution combines all weights used on held-out test folds including all temporal bins (for wheel-speed and wheel-velocity) and all repeated decoding runs, but excludes regression intercepts. The legend indicates the decoded variable, and the fraction of weights equal to zero (due to L1 regularization) is shown in parentheses.

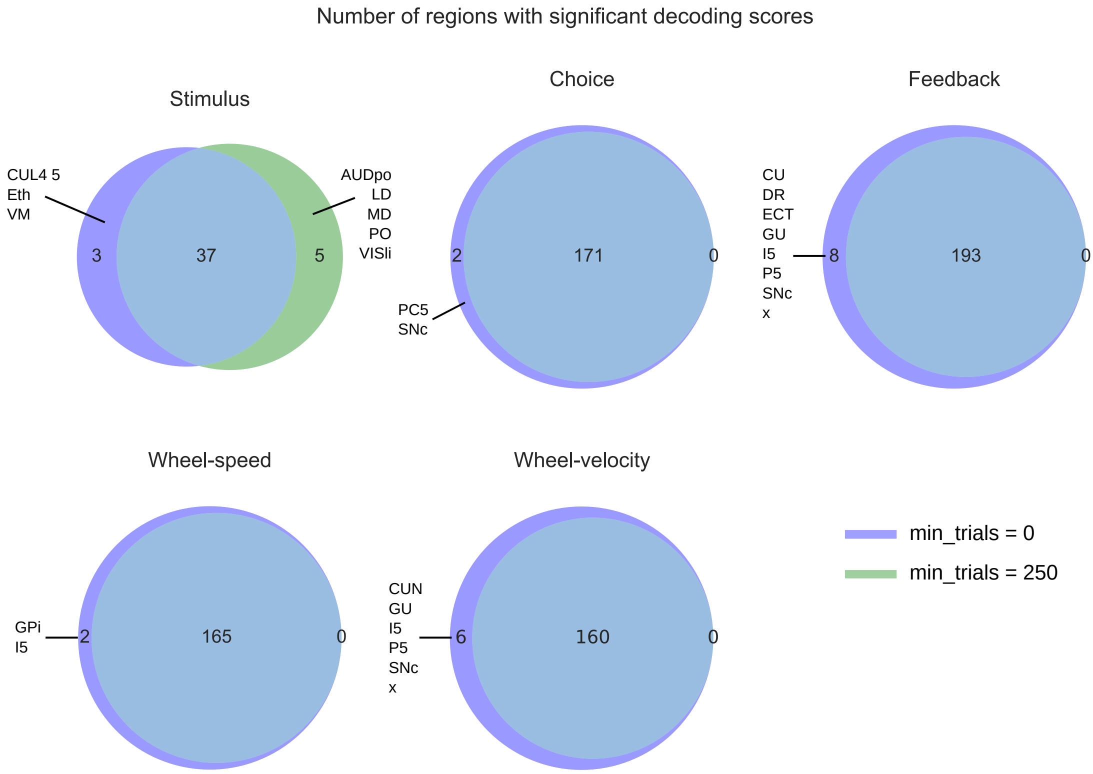

Figure S10. Number of regions with significant decoding scores when including a constraint on the minimum number of trials. The companion prior paper[117] requires a minimum of 250 trials in order to perform decoding of a given session. We waive that requirement for the decoding analyses in this paper in order to match the same neurons used in the other analyses, which affects the significance of only a small proportion of regions for each target variable.
